# Five-year clinical outcomes of a 2.25 mm sirolimus-eluting stent in Japanese patients with very small coronary artery disease: final results of the CENTURY JSV study

**DOI:** 10.1007/s12928-022-00890-y

**Published:** 2022-09-02

**Authors:** Koki Shishido, Kenji Ando, Yoshiaki Ito, Itaru Takamisawa, Junji Yajima, Takeshi Kimura, Kazushige Kadota, Shigeru Saito

**Affiliations:** 1 Department of Cardiology, Shonan Kamakura General Hospital, Okamoto 1370-1, Kamakura City, 247-8533 Japan; 2 Division of Cardiology, Kokura Memorial Hospital, Kokura, Japan; 3 Department of Cardiovascular Medicine, Saiseikai Yokohama City Eastern Hospital, Yokohama, Japan; 4 Department of Cardiology, Sakakibara Heart Institute, Tokyo, Japan; 5 Department of Cardiovascular Medicine, The Cardiovascular Institute Hospital, Tokyo, Japan; 6 Department of Cardiovascular Medicine, Graduate School of Medicine, Kyoto University, Kyoto, Japan; 7 Department of Cardiology, Kurashiki Central Hospital, Kurashiki, Japan

**Keywords:** Drug-eluting stent, Sirolimus, Bioresorbable polymer, Very small vessel

## Abstract

The aim of this study is to evaluate the long-term safety and efficacy of the 2.25 mm bioresorbable-polymer sirolimus-eluting Ultimaster stent in a Japanese patient population. Treatment of coronary artery disease in very small vessels is associated with an increased risk for cardiac events. The CENTURY JSV study is a prospective, multicenter, single-arm study. Seventy patients with stable and unstable coronary artery disease with a coronary lesion eligible for implantation with a 2.25 mm stent were enrolled in this study. Patients underwent clinical follow-up through 5-year after the PCI procedure. The mean age was 70.4 ± 9.2 years. The prevalence of diabetes mellitus was 37.1%, all not insulin dependent. The incidence of major adverse cardiac events, defined as cardiac death, target vessel myocardial infarction (MI), and clinically driven target lesion revascularization (CD-TLR) at 5 years was 5.7%. A non-Q wave MI was noted in 1.4% and 4.3% underwent a CD-TLR. There was no stent thrombosis during the entire follow-up period. No cardiac events were reported between 2 and 5 years. This is the first study to demonstrate safety and effectiveness for 5 years after treatment of very small coronary disease with 2.25 mm-diameter DES.

Clinical trial registration: UMIN000012928

## Introduction

The prevalence of small vessel treatment among patients undergoing a percutaneous coronary intervention (PCI) has been reported to be around 35–50% of the cases [1–4]. For the interventional cardiologist, small vessel PCI constitute a technical challenge as small vessels are more distally located in the coronary system with frequently a diffuse distribution of the atherosclerotic narrowing’s rather than a discrete lesion [5, 6]. Moreover, small vessel disease is associated with an increased risk for a cardiac event, including restenosis and stent thrombosis [2, 4]. The introduction of drug eluting stents (DES) has improved the outcomes versus bare metal stents due to the inhibition of in-stent neointimal proliferation resulting in a lower in-stent late lumen loss and so maintaining stent patency, even in small diameter stents which are more prone to restenosis [7, 8]. These promising outcomes and technical developments have started the development of stents suitable for the treatment of vessels < 2.5 mm. The 2.25 mm-diameter Ultimaster stent is an extension of the regular sizes that has been evaluated in a comprehensive clinical program [9, 10].

The current CENTURY JSV study was initiated to evaluate the safety and efficacy of the 2.25 mm-diameter Ultimaster stent and the 9-month angiographic and 2-year clinical outcomes have been reported earlier [11]. This update report has the final 5-year outcomes.

## Methods

### Study design and patient population

**Fig. 1:**
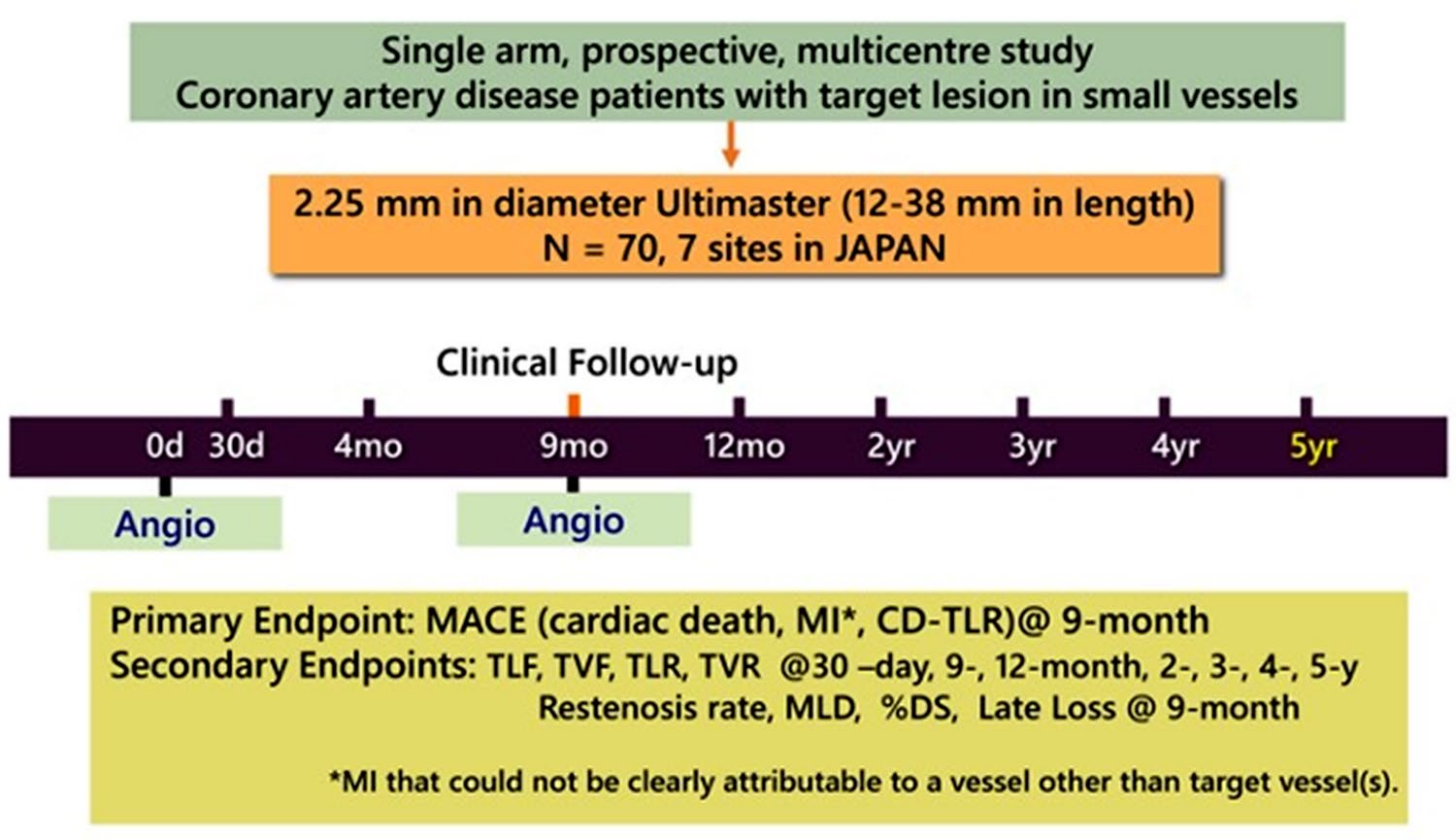
CENTURY JSV study design

The design of the CENTURY JSV study has been published earlier [11]. Briefly, it was a prospective, multicenter, single-arm study that enrolled patients with a minimum age of 20 years with asymptomatic myocardial ischemia, stable or unstable angina pectoris with a lesion suitable for implantation of a single 2.25 mm-diameter Ultimaster stent. Angiographic follow-up was performed at 9-month follow-up. Clinical follow-up was performed at 1 and 9 months, 1 year and annually up to 5 years (Fig. 1).

The target lesion had to be covered with one stent (maximum length 38 mm). Up to two non-target lesions could be treated during the index procedure provided that non-study stent implantation was uncomplicated before continuing the procedure with pre-dilatation of the target lesion. Patients with an acute myocardial infarction (MI) within 48 h before the procedure, renal failure requiring dialysis, left-ventricular ejection fraction < 25%, life expectancy < 1 year, or patients requiring a staged procedure were excluded. Anatomical exclusion criteria included bifurcation lesions requiring stenting of both main and side branch, ostial lesions, arterial and venous bypass grafts, left main lesions, in-stent restenosis, and lesion requiring preparation other than balloon pre-dilation. The study was performed according to the Declaration of Helsinki and Good Clinical Practice, and all patients provided written informed consent. The Institutional Review Board of each participating site approved the study. The UMIN-CTR clinical trial registration number is UMIN000012928.

### Study device

The Ultimaster stent (Terumo Corporation, Tokyo, Japan) is a thin-strut (80 μm) cobalt–chromium sirolimus-eluting stent with an open-cell design [9]. Sirolimus is embedded in a biodegradable polymer coating (poly-D, L-lactic acid polycaprolactone) that is fully metabolized through dl-lactide and caprolactone into carbon dioxide and water in 3–4 months. The gradient coating reduces polymer cracking and delamination at the stent hinges. The coating is applied on the abluminal side of the struts only, and a bare metal stent remains after resorption of the coating in 3–4 months. For the current study, stent lengths of 12, 15, 18, 24, 28, 33, and 38 mm were available with a diameter of 2.25 mm.

### Procedural and post-interventional practices

Apart from mandatory lesion pre-dilatation, PCI was performed in accordance with the standard procedure of each hospital. Lesion preparation with other devices than pre-dilatation balloons was an exclusion criterion. The aim was to cover the lesion with a single stent. In case of a bail-out situation, implantation of additional stents was allowed. Post-dilatation was at the discretion of each operator. Antiplatelet therapy with aspirin and a P2Y12 inhibitor was started before the index procedure and maintained for a minimum of 9 months.

### Follow-up, study endpoints, and definitions

The primary endpoint was the incidence of major adverse cardiac events (MACE) at 9 months, defined as cardiac death, target vessel MI, and clinically driven target lesion revascularization (TLR). Cardiac death was defined as any death due to proximate cardiac cause (e.g., MI, low-output failure, fatal arrhythmia), unwitnessed death and death of unknown cause, and all procedure-related deaths, including those related to concomitant treatment. MI was defined either as the development of pathological Q-waves in at least two contiguous leads with or without elevated cardiac enzymes or, in the absence of pathological Q-waves, as an elevation in creatinine kinase levels to greater than twice the upper limit of normal in the presence of an elevated level of CK-MB fraction or troponin. Cardiac biomarkers were obtained before the procedure and after the procedure in intervals of 6 h up to 24 h to detect myocardial injury. TLR was defined as repeat percutaneous intervention of the stented lesion including 5 mm proximal and distal from the edge of the stent, or bypass surgery of the target vessel that was performed for a clinical indication and was due to restenosis or closure of the target lesion. A revascularization was considered clinically indicated if prompted by a positive functional study, or ischemic ECG changes at rest in a distribution consistent with the target vessel, or ischemic symptoms with an in-lesion diameter stenosis ≥ 50% by QCA or if lesion diameter stenosis was more than 70% at follow-up, even in the absence of clinical symptoms. Target lesion failure (TLF) was defined as cardiac death that cannot be clearly attributed to a vessel other than the target vessel, target vessel MI, and clinically driven target lesion revascularization. Target vessel failure (TVF) was defined as cardiac death that cannot be clearly attributed to a vessel other than the target vessel, target vessel MI, and clinically driven target vessel revascularization. Stent thrombosis and bleedings were defined by the Academic Research Consortium and Bleeding Academic Research Consortium (BARC) definitions, respectively [12, 13]. Stent thrombosis was picked up definite, probable and possible stent thrombosis. Bleeding events were picked up only BARC 3 and 5 bleeding. All events were adjudicated by a Clinical Events Committee.

### Statistical analysis

Continuous variables are reported as means along with the standard deviation (SD). Categorical variables are reported as frequencies and percentages. The cumulative event free rates as a function of time were calculated by the Kaplan-Meier method. All the analyses were carried out using SAS version 9.4 (SAS Institute, Japan Ltd.).

## Results

### Baseline and procedural characteristics

The study enrolled 70 patients between April 16 and December 25, 2014 by seven sites in Japan. The enrolled patients represented a typical population requiring a PCI with a mean age of 70.4 ± 9.2 years and a predominantly male gender (77.1%). The prevalence of diabetes mellitus was 37.1%, but none of the patients required insulin treatment. Regarding other classic cardiovascular risk factors, hypertension and dyslipidemia were present in 87.1% of the patients. Previous smoking was reported by 42.9% of the patients; 11.4% of the patients was a current smoker. A previous PCI was performed in 52.9%. Table 1 shows an overview of the co-morbidities. The location of the target lesions was the left circumflex in 42.9% and the left anterior descendants in 32.9% of the patients. A bifurcation was involved in 21.4%. The mean lesion length was 14.6 ± 7.6 mm, and the mean reference vessel diameter was 2.0 ± 0.3 mm. In total, 72 Ultimaster 2.25 mm stents were implanted; two (2.9%) patients required an extra study stent because of a bail-out situation. The mean length of the implanted Ultimaster stents was 21.4 ± 8.2 mm. Post-dilatation was performed in 75.7% resulting in a mean post procedural in-stent diameter stenosis of 11.7 ± 8.7%. Summary of lesion and procedural characteristics are shown in Table 2.

**Table 1:** Patient demographics

Number of patients	70
Age (Mean ± SD)	70.4 ± 9.2
Gender, males (%)	54(77.1)
Type of angina (%)	
Stable	61(87.1)
Unstable	4(5.7)
Silent ischemia	5(7.1)
Diabetes (%)	26(37.1)
IDDM (%)	0(0)
Hypertension (%)	61(87.1)
Dyslipidemia (%)	61(87.1)
Cerebrovascular disease (%)	2(2.9)
Peripheral artery disease (%)	5(7.1)
Congestive heart failure (%)	3(4.3)
Family history of CAD (%)	19(27.1)
smoked in the past (%)	30(42.9)
Current smoker (%)	8(11.4)
Previous PCI (%)	37(52.9)
Previous CABG (%)	0(0)
Previous MI (%)	19(27.1)
Previous Stroke (%)	8(11.4)

**Table 2:** Lesion/procedural characteristics

Number of lesions	70	
Target vessel location (%)		
LMT	0(0)	
LAD	23(32.9)	
Proximal	1 (1.4)	
Mid	7 (10.0)	
Distal	15 (21.4)	
LCx	30(42.9)	
Proximal	0 (0)	
Mid	8 (11.4)	
Distal	22 (31.4)	
RCA	17(24.3)	
Proximal	1 (1.4)	
Mid	3 (4.3)	
Distal	13 (18.6)	
Lesion classification (%)		
A	6(8.6)	
B1	16(22.9)	
B2	25(35.7)	
C	23(32.9)	
Bend(> 45 deg.) (%)	6(8.6)	
Calcification* (%)	5(7.1)	
Tortuosity* (%)	17(24.3)	
Bifurcation (%)	15(21.4)	
Stent length (mean ± SD) (mm)	21.4 ± 8.2	
Post-dilatation (%)	53(75.7)	
Overlapping (%)	2(2.9)	
%DS < 30 after PCI (visually) (%)	69(98.6)	
Angiographic measurement		
Before procedure		
Lesion length (mm)	14.64 ± 7.58	
Reference vessel diameter (mm)	1.95 ± 0.28	
Minimal lumen diameter (mm)	0.67 ± 0.23	
Diameter stenosis (%)	65.5 ± 9.9	
After procedure	In stent	In segment
Minimal lumen diameter (mm)	1.99 ± 0.27	1.57 ± 0.38
Diameter stenosis (%)	11.7 ± 8.7	29.7 ± 11.7
Acute gain (mm)	1.31 ± 0.29	0.89 ± 0.36
*: moderate + severe		

### Clinical outcomes

**Fig. 2:**
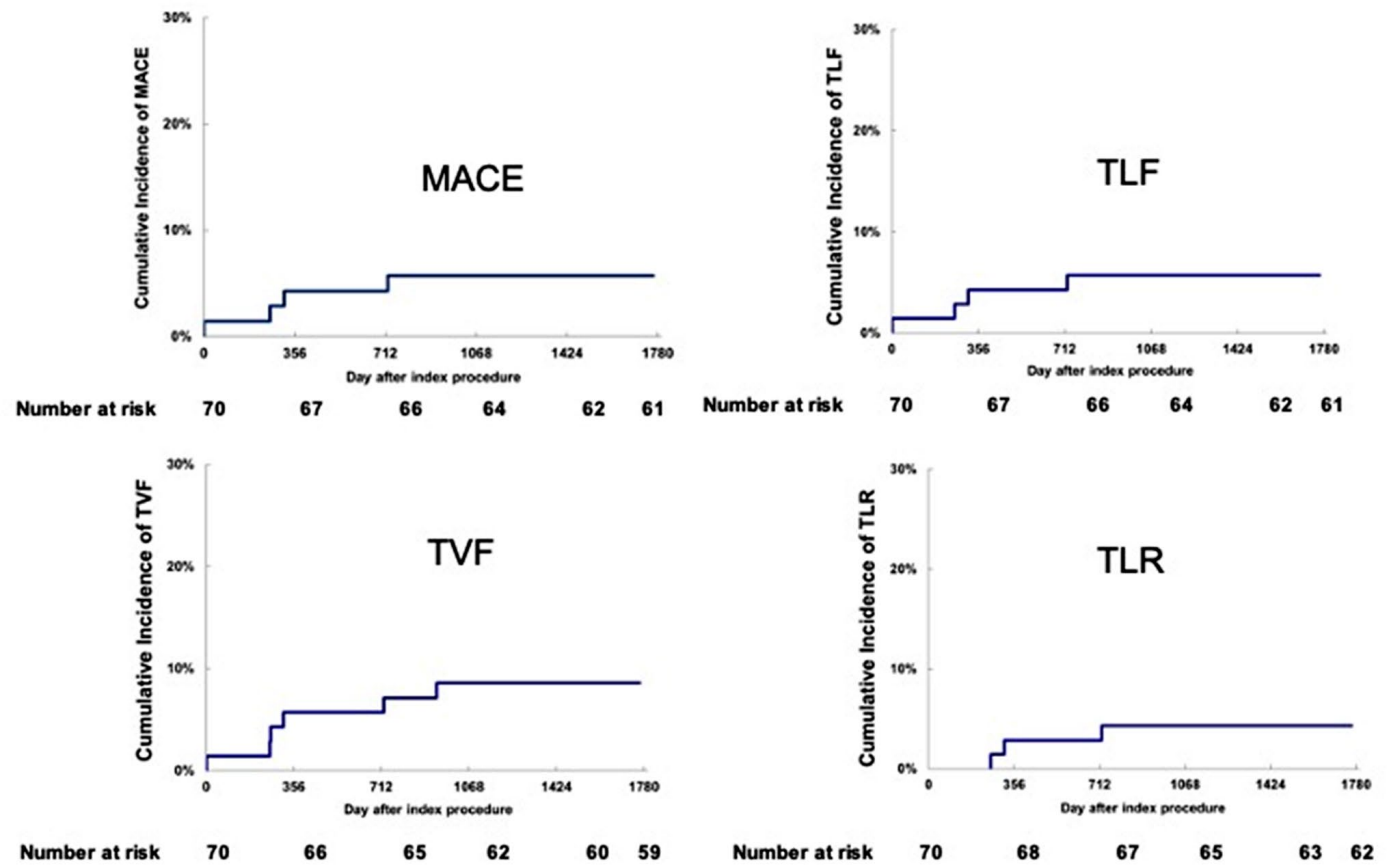
Kaplan–Meier curve of MACE, TLF, TVF, and TLR rate up to 5 years after implantation of Ultimaster φ2.25 mm stent

All patients were followed up to 5 years except 4 patients who died due to non-cardiac causes. An MACE was observed in 4 patients (5.7%) throughout 5 years. No cardiac death was reported. One patient experienced non-Q MI and three patients underwent a clinically driven TLR. All these events occurred before the 2-year follow-up. None of the patients reported an MACE between 2 and 5 years. Furthermore, there were no instances of stent thrombosis (as defined by the Academic Research Consortium) through 5 years. TLF and TVF were observed in 4 patients (5.7%) and 6 patients (8.6%) at 5 years, respectively. Bleeding evens were observed in 3 patients (4.3%) at 5 years after the procedure (Table 3). Kaplan–Meier curves for each clinical event are drawn in Fig. 2.

**Table 3:** Clinical outcomes at 9 months, and 1 to 5 years

	9 months	1 year	2 years	3 years	4 years	5 years
	(*N* = 70)	(*N* = 70)	(*N* = 70)	(*N* = 70)	(*N* = 70)	(*N* = 70)
Death	0 (0%)	0 (0%)	0 (0%)	1 (1.4%)	3 (4.3%)	4 (5.7%)
Any cause	0 (0%)	0 (0%)	0 (0%)	1 (1.4%)	3 (4.3%)	4 (5.7%)
Cardiac	0 (0%)	0 (0%)	0 (0%)	0 (0%)	0 (0%)	0 (0%)
MI	1 (1.4%)	1 (1.4%)	1 (1.4%)	1 (1.4%)	1 (1.4%)	1 (1.4%)
Q MI	0 (0%)	0 (0%)	0 (0%)	0 (0%)	0 (0%)	0 (0%)
Non -Q MI	1 (1.4%)	1 (1.4%)	1 (1.4%)	1 (1.4%)	1 (1.4%)	1 (1.4%)
Clinically driven TLR	1 (1.4%)	2 (2.9%)	3 (4.3%)	3 (4.3%)	3 (4.3%)	3 (4.3%)
Clinically driven TVR	2 (2.9%)	3 (4.3%)	4 (5.7%)	5 (7.1%)	5 (7.1%)	5 (7.1%)
MACE	2 (2.9%)	3 (4.3%)	4 (5.7%)	4 (5.7%)	4 (5.7%)	4 (5.7%)
Cardiac Death	0 (0%)	0 (0%)	0 (0%)	0 (0%)	0 (0%)	0 (0%)
Q MI	0 (0%)	0 (0%)	0 (0%)	0 (0%)	0 (0%)	0 (0%)
Non -Q MI	1 (1.4%)	1 (1.4%)	1 (1.4%)	1 (1.4%)	1 (1.4%)	1 (1.4%)
Clinically driven TLR	1 (1.4%)	2 (2.9%)	3 (4.3%)	3 (4.3%)	3 (4.3%)	3 (4.3%)
TLF	2 (2.9%)	3 (4.3%)	4 (5.7%)	4 (5.7%)	4 (5.7%)	4 (5.7%)
TVF	3 (4.3%)	4 (5.7%)	5(7.1%)	6 (8.6%)	6 (8.6%)	6 (8.6%)
Stent Thrombosis	0 (0%)	0 (0%)	0 (0%)	0 (0%)	0 (0%)	0 (0%)
Bleeding and vascular complication	0 (0%)	0 (0%)	0 (0%)	2 (2.9%)	3 (4.3%)	3 (4.3%)
Stent fracture	0 (0%)					

*MI;* MI that cannot be clearly attributed to a vessel other than the target vessel, *TLF;* cardiac death that cannot be clearly attributed to a vessel other than the target vessel, target vessel MI, and clinically driven target lesion revascularization, *TVF;* cardiac death that cannot be clearly attributed to a vessel other than the target vessel, target vessel MI, and clinically driven target vessel revascularization

## Discussion

To the best of our knowledge, this is the first study to demonstrate the 5-year safety and efficacy of very small vessel PCI with 2.25-mm DES. In detail, there were no cardiac death, one (1.4%) non-Q wave MI, and three (4.3%) clinically driven TLRs. All events occurred in the first 2 years; no cardiac events were reported between 2 and 5 years. Notably, there were no stent thrombosis during the entire follow-up period.

Coronary vessel diameter has a continuous inverse relationship with procedural complexity and the incidence of cardiac events [4, 14, 15]. Correspondingly, the definitions of what constitute a small vessel is arbitrary, varies between studies and has evolved over time [6]. Initially, in the early days of bare metal stents, vessels ≤ 2.9 mm in lumen diameter were considered small [16]. Over time, with the introduction of DES, the upper threshold has been lowered to 2.75 or 2.5 mm, or even lower depending on the trial [4, 17]. Based on cardiac event rates with novel DES, a vessel diameter of 2.5 mm has been suggested to identify small target vessels [2]. The advent of DES technology with small strut designs as well as the availability of low-profile delivery balloons allowed the design of 2.0- and 2.25-mm stent iterations to treat very small vessels and introduced corresponding terminology to distinguish these vessels from the ‘regular’ small vessels [8, 11, 18].

The therapeutic options for (very) small vessels are similar as for all coronary artery disease and include medical therapy and revascularization by PCI or coronary artery bypass grafting (CABG). Guidelines recommend CABG for patients with multivessel disease and higher Syntax scores [19, 20]. However, small vessel disease, often characterized by diffuse atherosclerosis over a longer segment of the distal vessel, constitutes a technical challenge to anastomose the graft with sufficient run-off with consequently an increased risk for an incomplete revascularization and cardiac events [21]. Similarly, a distal lesion can be difficult to reach for a PCI, and diffuse disease over longer segments in multiple vessels reduces the chance for a successful procedure [3]. In addition, small vessel disease remains an independent predictor of MACE after PCI [22]. Careful decision-making by the Heart Team, weighing the pros and cons of the therapeutic options including the patient’s preference, is the cornerstone of contemporary patient management for patients with challenging anatomies such as small vessel disease.

The result of the current study demonstrates that PCI is a suitable treatment option for selected patients with very small vessel disease. All stents were successfully implanted, and the peri-procedural cardiac event rate (1.4%) was low. During the 5-year follow-up period, no MACE events occurred after 2 years resulting in a final low MACE rate of 5.7%. Ultimaster stents are designed, such that their polymers (poly DL-lactic acid) are absorbed over the course of 3–4 months. After that, the polymer was completely dissolved, and the less inflammatory bare metal surface came into direct contact with the vascular wall, accommodating relatively healthy and homogeneous neointimal tissue growth for the remainder. In addition, Wilson et al. [23] demonstrated that the use of a bioresorbable-polymer coating as a method for drug elution results in lower long-term inflammation compared to durable polymer DES. Furthermore, Itoh et al. [24] showed that qualitatively and quantitatively consistent neointimal stent coverage was achieved by the 12-month time point by optical frequency-domain imaging. After the polymer was completely dissolved, and the less inflammatory bare metal surface came into direct contact with the vascular wall, accommodating relatively healthy and homogeneous neointimal tissue growth. These reactions might affect the absence of MACE and TLR from 2 years on.

A clinically driven TLR rate of 4.3% is impeccable, as even small amounts of intimal hyperplasia can cause functional or anatomical restenosis within a narrow stent lumen. The in-stent late lumen loss of 0.22 ± 0.31 mm and the in-stent binary restenosis rate of 4.3% at 9 months earlier reported for this study are re-assuring in that perspective and provide an angiographic substantiation for the clinical outcomes [11].

### Comparison with the study results for small vessels conducted in the past

Kandzari et al. [25] reported that cardiac death or MI in the small vessel (≤ 2.25 mm) subgroup of the PROMUS Element Plus US Post-Approval Study was 13% and TLF was 16%. Pilgrim et al. [26] reported in a BIOSCIENCE randomized trial that the TLFs of BP-SES and DP-SES for small vessels (defined as stent diameter in any lesion ≤ 3 mm) were 20.3% and 18.4% (P = 0.241), respectively. Lefèvre et al. [27] reported in BIO FLOW-II that the TLFs of O-SES and X-EES for small vessels (≤ 2.75 mm) were 11.1% and 15.5% (P = 0.303), respectively. Kelly et al. [28] reported that the TLF of Pt-Cr EES for small vessels (diameter < 2.5 mm) was 7.0% in the PLATINUM Trial. Event rates were above 10% in most trials. These results show that even when DES are used, treatment of very small vessels can still be challenging depending on patient and lesion-specific characteristics.

Similar to our study, Saito et al. [29] reported 4-year results of the RESOLUTE Small Vessel Study that evaluated Resolute in Japanese patients (RESOLUTE Japan SV study). There were some differences of patient and lesion characteristics between CENTURY JSV study and RESOLUTE Japan SV study. Rate of IDDM was significantly higher in RESOLUTE Japan SV study (10.8 vs. 0%); in the other hand, ratio of B2 and C in lesion classification was higher in CENTURY JSV study (68.6 vs. 45.1%). There is no difference in other characteristics. The MACE rate at 2 years of RESOLUTE Japan SV was 5.4% but increased over time to 12.3% at 4 years. Similarly, the TLF of the small vessel group of PERSEUS SV Trial and BIOFLOW-II also increased over time. Konigstein et al. [15] demonstrated that reference vessel diameter was the only lesion-related predictor of long-term TLF from individual patient data pooled analysis from 6 randomized controlled trials. The reference vessel diameter of the treated vessels in our study was very small (1.95 ± 0.28 mm). Despite implanting in such small vessels, it is a remarkable result that the 5-year MACE was extremely low at 5.7%; additionally, there is no subsequent occurrence from 2 year follow-up.

### Comparison between drug-coated balloon (DCB) and DES in small vessels

Regarding strategy for native small vessel disease, there is conflicting evidence about the effects of DCB compared with DES in patients with native small vessel disease. The PICCOLETO study which randomized between first-generation DCB and DES reported a trend toward higher major adverse cardiovascular events rate at 9 months in the DCB group [30]. On the other hand, new-generation DCB studies showed that DCB use was associated with comparable risk of TLR and MACE in the BELLO study [31], RESTORE study [32], and BASKET-SMALL 2 study [33]. The recent PICCOLETO II trial showed that new-generation DCB was found superior to DES in terms of late lumen loss by angiographic follow-up [34]. However, the limitations of these trial were not large number of patients and not powered for clinical endpoints. An SCAAR report, including the largest real-world population with small vessel disease, showed that DCB was associated with approximately double the risk for restenosis at long-term follow-up compared with new-generation DES even after propensity score matching for baseline characteristics [35]. DCB use should be therefore limited to selected cases for which stent implantation is not desirable.

### Strengths and limitations

The study was designed as a single-arm study and comparison of outcomes with other treatment options is limited by inherent design differences between studies. A single stent designed for very small vessels was evaluated and the results can therefore not be extrapolated to other stent models. The angiographic evaluation at 9 months allows to correlate these findings with the clinical outcomes. The 5-year follow-up is sufficiently long to assess the long-term outcomes. The number of patients is low and has been selected according to protocol criteria limiting the generalization to a larger population of patients with small vessel disease.

## Conclusions

The final 5-year results of the CENTURY JVS study establish the long-term safety and effectiveness of the 2.25 mm Ultimaster bioresorbable-polymer sirolimus-eluting stent for the treatment of very small coronary disease in Japanese patients.

## Abbreviations

DES: Drug-eluting stent
MACE: Major adverse cardiac events
MI: Myocardial infarction
PCI: Percutaneous coronary intervention
TLR: Target lesion revascularization
TLF: Target lesion failure
TVF: Target vessel failure
